# Serious infection incidence rates in pediatric systemic lupus erythematosus according to medication use

**DOI:** 10.1186/ar4655

**Published:** 2014-09-18

**Authors:** Linda T Hiraki, Candace H Feldman, Mary Beth Son, Jessica M Franklin, Michael A Fischer, Daniel H Solomon, Seoyoung C Kim, Wolfgang C Winkelmayer, Karen H Costenbader

**Affiliations:** 1 The Hospital for Sick Children, Toronto, ON, Canada; 2 Brigham and Women's Hospital, Boston, MA, USA; 3 Boston Children's Hospital, Boston, MA, USA; 4 Stanford University School of Medicine, Stanford, CA, USA

## Background

We investigated incidence rates of serious infections among children with systemic lupus erythematosus (SLE) and lupus nephritis (LN) enrolled in Medicaid, the US health insurance program for low-income children and parents.

## Methods

We identified all children aged 5 to <18 years with SLE (≥3 ICD-9 codes of 710.0, each >30 days apart) and LN (≥2 ICD-9 codes for renal disease on/after SLE diagnosis) in the Medicaid Analytic eXtract (MAX) from 2000 to 2006. This dataset contains all outpatient and inpatient Medicaid claims for enrollees in 47 US states and the District of Columbia. Filled prescriptions were documented and patients were classified as new users of hydroxychloroquine (HCQ), corticosteroids (CS) and immunosuppressants (IS). We identified serious infections from hospital discharge diagnosis codes for all infections, and for specific subtypes of infections (bacterial, fungal and viral). We calculated incidence rates per 100 person-years (PY) overall and by medication subgroup. Incidence rate ratios (IRR) (95% CI) were calculated comparing CS, IS and CS+IS with HCQ alone using Poisson models, adjusted for age, sex and duration of enrollment in Medicaid.

## Results

Among the 2,403 children with SLE who were new medication users, there were 316 serious infections in 2,215 PY. Incidence rates for all serious infections requiring hospitalization varied between 10/100 PY for SLE and 25.7/100 PY for LN. Among children with SLE receiving CS alone, incidence rates of serious infections were 3.5 times higher compared with those not receiving CS or IS. Among children receiving both CS and IS, overall serious infection incidence rates were twice as high compared with children not receiving either medication (Table 1).

**Table 1:** 

	Systemic lupus erythematosus		Lupus nephritis	
	
	Unadjusted IRR (95%CI)	MV-adjusted^a ^IRR (95% CI)	Unadjusted IRR (95%CI)	MV-adjusted^a ^IRR (95% CI)
HCQ	Reference	Reference	Reference	Reference
CS	**5.25 (4.07, 6.78)**	**3.58 (2.79, 4.59)**	**2.13 (1.51, 3.01)**	**1.90 (1.36, 2.67)**
IS	**2.58 (1.86, 3.58)**	**2.46 (1.80, 3.37)**	1.28 (0.80, 2.05)	1.12 (0.70, 1.78)
CS+IS	**2.90 (2.03, 4.14)**	**1.99 (1.41, 2.80)**	NR	NR

NR, not reported in accordance with CMS policy. ^a^Multivariable adjusted models included age, sex, race/ethnicity, US residential region, area-level SES, SLE severity index.

## Conclusions

Infection remains a common complication of SLE and is associated with significant morbidity and mortality. We observed high rates of serious infections requiring hospitalization among children with SLE and LN. Those children receiving CS alone or in combination with IS had much higher rates of serious infections, compared with those children receiving HCQ alone.

